# The Association Between Dietary Diversity Score and Odds of Diabetic Nephropathy: A Case-Control Study

**DOI:** 10.3389/fnut.2022.767415

**Published:** 2022-04-01

**Authors:** Mahsa Rezazadegan, Fatemeh Mirjalili, Yahya Jalilpiran, Monireh Aziz, Ahmad Jayedi, Leila Setayesh, Mir Saeed Yekaninejad, Krista Casazza, Khadijeh Mirzaei

**Affiliations:** ^1^Department of Clinical Nutrition, School of Nutrition and Food Science, Food Security Research Center, Isfahan University of Medical Sciences, Isfahan, Iran; ^2^Department of Clinical Nutrition, School of Nutritional Science and Dietetics, Tehran University of Medical Sciences, Tehran, Iran; ^3^Students' Scientific Research Center, Tehran University of Medical Sciences, Tehran, Iran; ^4^Department of Clinical Nutrition, School of Nutrition and Food Sciences, Shiraz University of Medical Sciences, Shiraz, Iran; ^5^Social Determinants of Health Research Center, Semnan University of Medical Sciences, Semnan, Iran; ^6^Department of Community Nutrition, School of Nutritional Science and Dietetics, Tehran University of Medical Sciences, Tehran, Iran; ^7^Department of Epidemiology and Biostatistics, School of Public Health, Tehran University of Medical Sciences, Tehran, Iran; ^8^Marieb College of Health and Human Services, Florida Gulf Coast University, Florida, FL, United States

**Keywords:** diet variety, diabetic nephropathy, case-control, Iran, dietary diversity score

## Abstract

A dietary diversity score (DDS) may be a useful strategy for monitoring risks associated with chronic diseases. Few studies have investigated the relationship between DDS and the progression to chronic kidney disease (CKD). A better understanding of the relationship between DDS and diabetic nephropathy (DN) may provide insight for monitoring the overall diet and clinical outcomes. This case-control study included 105 women with DN and 105 controls with age and diabetes duration-matched to evaluate the extent to which DDS is associated with DN. Dietary intake was assessed using the food frequency questionnaire (FFQ). DDS was calculated based on the method using five food groups: bread/grains, vegetables, fruits, meats, and dairies. Conditional logistic regression was performed to examine the association between DDS and odds of DN. Anthropometric measures and physical activity levels were evaluated using standard protocols. In a fully adjusted model [controlled for age, body mass index (BMI), energy intake, physical activity, diabetes duration, cardiovascular disease history, and drug usage], greater adherence (the third vs. the first tertile) to DDS [odds ratio (OR) = 0.13; 95% CI (0.05–0.35)], vegetables group [OR = 0.09; 95% CI (0.02–0.36)], and fruits group [OR = 0.05; 95% CI (0.01–0.20)] were significantly associated with lower odds of DN. However, we did not observe any significant relationship between other DDS components and the odds of DN. Our findings showed that higher DDS might be associated with reduced odds of DN. However, more prospective studies are warranted to confirm these findings.

## Introduction

The increasing burden of type 2 diabetes (T2D) is a concern in the healthcare system worldwide (1). The diabetes prevalence is estimated to rise from 9.3 to 10.9% between 2019 and 2045 globally (2). The significant mortality rate observed in T2D is profoundly increased by the co-occurrence of diabetic nephropathy (DN) affecting ~40% of patients with T2D (3). The most common indicator for the diagnosis of DN is albuminuria (urinary albumin to creatinine ratio ≥ 30 mg/g) and also a reduction in the estimated glomerular filtration rate (eGFR) (<60 ml/min/1.73 m^2^) (4). Previous studies reported that DN occurs about 10–20 years after the onset of T2D (5). Some of the major risk factors for DN, such as ethnicity and family history, are non-modifiable. However, hypertension, gestational diabetes, insulin resistance, obesity, hyperlipidemia, and increased glycosylated hemoglobin levels can each be mitigated by dietary modification (6). Consideration of nutritional approaches in addition to pharmacological therapy is an important strategy to prevent the onset and progression of DN (7). The diet of patients with chronic kidney disease (CKD) needs to provide healthy food options to enhance their diet quality (8). Accordingly, previous studies showed that restriction of protein, phosphorus, and sodium intake can improve renal function in patients with DN (9).

A recent observational study suggested that adherence to the Dietary Approaches to Stop Hypertension (DASH) diet was inversely associated with a risk of end-stage renal disease (ESRD) in adults with CKD, particularly, in diabetic persons (10). Dietary diversity score (DDS) is an indicator for assessing the diet quality and nutrient adequacy ratio (11, 12). Previous studies have identified a relationship between DDS and some risk factors for diabetes and metabolic syndrome, including, hyperglycemia, adverse lipid profiles, and low serum adiponectin level (13–15). A recent cross-sectional study in Bangladesh demonstrated that the serum creatinine level of newly diagnosed T2D was negatively correlated with the DDS (16). Furthermore, renal transplant patients with higher dietary diversity had a lower prevalence of obesity (17). The present case-control study is aimed to examine the association between DDS and the odds of DN among women with T2D in Semnan, Iran.

## Materials and Methods

### Participants

This case-control study was conducted in the Kowsar Diabetes Clinic, Semnan, Iran, from July to December 2016. A total of 210 women (105 cases and 105 controls) participated in this study. Inclusion criteria were women with prevalent T2D, aged between 30 and 65 years, and with a T2D diagnosis of 3–10 years. The American Diabetes Association's most recent diagnostic criteria (18) were used to define diabetes in this study. Established cut-points were fasting blood glucose (FBG) ≥ 126 mg/dl, 2-h post-load blood glucose (2hrBG) ≥ 200 mg/dl, and hemoglobin A1c (HbA1c) ≥ 6.5%. Participants with autoimmune disorders, history of cancer, coronary angiography, hepatic disease, myocardial infarction, and stroke were excluded. Urinary albumin-to-creatinine ratio (ACR) ≥ 30 mg/g in a random spot urine sample was considered as DN (19). From chart review, 120 patients with DN were identified. However, only 105 patients consented to participate. In total, 105 diabetic women without DN from the same center as the control group were matched for age and the duration of diabetes with the case group. Incomplete data in the food frequency questionnaire (FFQ), implausible responses, and total energy intake of <500 or >3,500 kcal/day were considered as exclusion criteria (20). All participants provided informed consent. This research was approved by the Ethics Committee of Tehran University of Medical Sciences (Ethics number: IR.TUMS.REC.1395.2644) and the Ethics Committee of Semnan University of Medical Sciences (Ethics number: IR. SEMUMS. REC.1395.66).

### Demographic, Anthropometric, and Blood Biomarkers Assessment

Demographic data, including age, diabetes duration, physical activity, history of cardiovascular disease, and current drug usage, were collected by trained interviewers. Bodyweight (kg) was measured while subjects wore light clothing and no shoes. Height was measured at 0.1 cm precision by a non-stretchable tape in a standing position without shoes. Body mass index (BMI) was calculated as weight in kilograms divided by the square of height in meters. Systolic blood pressure (SBP) and diastolic blood pressure (DBP) were measured once on the left arm while sitting after a resting period of ≥5 min using a manual sphygmomanometer.

To assess subjects' physical activity, we used a standard physical activity questionnaire (IPAQ) (21). Scoring criteria based on this questionnaire indicated “low physical activity” (score <600 Metabolic Equivalents/Week), “moderate physical activity” (score between 600 and 3,000 MET/h/Week), and “high physical activity” (score > 3,000 MET/h/Week). Biochemical indexes, including fasting blood sugar (FBS), 2hrBG, HbA1c, total cholesterol (TC), triglycerides (TG), low-density lipoprotein (LDL), high-density lipoprotein (HDL), total serum creatinine (Cr), and blood urea nitrogen (BUN), were obtained from participant's medical records during the past 3 months.

### Dietary Intake Assessment and DDS Calculation

A food-frequency questionnaire found to be valid and reliable in this population was used to assess dietary intake (22). This FFQ consisted of a list of 147 food items with standard serving sizes commonly used by Iranians. Participants reported the frequency of consumption of a given food item during the previous year. Participants reported their intake of food or food items daily, weekly, monthly, or yearly. Final portion sizes were changed into g/day using household measures. Then, these amounts were adjusted for energy intake using the residual method (23). Dietary intakes were analyzed using Nutritionist IV (First Data Bank, San Bruno, CA, USA) software to estimate energy and nutrient intakes.

For scoring dietary diversity, we used a method described by Kant et al. (24, 25). This method was based on five groups, including grains, vegetables, fruits, meats, and dairy products, according to the United States Department of Agriculture (USDA) food guide pyramid. Before computing the DDS, we adjusted all food groups for energy intake. Although the Kant method includes seven subgroups for grains, the method employed in this study used five subgroups, refined bread, macaroni, corn flakes, rice, and biscuits. Other subgroups were aligned with the Kant method, such that the vegetable group was divided into mixed vegetables, potato, tomato, other starchy vegetables, legumes, yellow vegetables, and green vegetables. The fruits group was composed of fruit and fruit juice, berries, and citrus fruits. The group of meat included four subgroups, such as red meat, poultry, fish, and eggs. Eventually, the group of dairy products was defined as milk, yogurt, and cheese. A maximum score of 2 was considered for each food group. Therefore, the total DDS was in the range of 0–10.

Subjects were considered as a “consumer” who score 1 for any component of food groups if they had intaken higher than median amounts; otherwise, they were allocated a score of 0. Afterward, the sum of components' scores in each food group was computed to have a total score of the related food group. The total scores in each group were divided into the number of components in that group. Then, the aforesaid value was multiplied by 2. The total DDS for each subject was estimated by summing up the figures for food groups. For instance, if a subject had dietary intakes of whole-grain bread, rice, and macaroni higher than the median amounts, his or her score was calculated as (3/6) × 2 = 1.0. After computing the DDS for the other four groups, total DDS was determined. Hence, the total score of dietary diversity for each participant was between 0 (minimum) and 10 (maximum) points.

### Statistical Analysis

The normality of quantitative variables was assessed using the Kolmogorov-Smirnov test. General characteristics among cases and controls were compared using paired sample *t*-test and chi-square or Fischer's exact tests. We used one-way ANOVA and chi-square tests to compare quantitative variables across the tertiles of DDS and to determine the distribution of the qualitative variables across the tertiles of DDS, respectively. Energy-adjusted dietary macro- and micronutrient intakes were also compared across the tertiles of DDS using the analysis of covariance (ANCOVA) test. Conditional logistic regression was performed to estimate the associations between the DDS and the odds of DN. In adjusted models, age, BMI, energy intake, physical activity, diabetes duration, cardiovascular disease history, and pharmacotherapy [angiotensin receptor blockers (ARBs); angiotensin-converting enzyme inhibitors (ACIE), beta-blockers, metformin, sulphonylurea, and insulin] were controlled. SPSS software (version 25, SPSS Inc., Chicago, IL, USA) was used for data analysis, with *p* < 0.05 was considered statistically significant.

## Results

Sociodemographic characteristics and anthropometric measures of study participants are displayed in Table 1. Groups were similar except for using ACIE drugs, which were more prevalent in cases than controls (*p* = 0.001).

**Table 1:** Sociodemographic characteristics and anthropometric measures of study participants.

**Variables**	**Cases** **(*N* = 105)**	**Controls** **(*N* = 105)**	***p* (paired *t*-test)**
Age (year)	*55.3 (7.0)*	*55.4 (7.1)*	0.94
Body mass index (kg/m^2^)	*28.7 (4.7)*	*27.5 (4.4)*	0.06
Diabetes duration (years)	*7.6 (2.2)*	*7.6 (1.1)*	0.88
Physical activity			0.13
Low	31 (29.5)	37(35.2)	
Moderate	42 (40.0)	28 (26.7)	
High	32 (30.5)	40 (38.1)	
History of cardiovascular disease			1.00
Yes	24 (22.9)	23 (21.9)	
No	81 (77.1)	82 (78.1)	
Angiotensin receptor blockers drug usage			0.05
Yes	60 (57.1)	45 (42.9)	
No	45 (42.9)	60 (57.1)	
Angiotensin converting enzyme inhibitors drug usage			0.001
Yes	44 (41.9)	21 (20.0)	
No	61 (58.1)	84 (80.0)	
Beta blockers drug usage			0.72
Yes	20 (19.0)	18 (17.1)	
No	84 (80.0)	87 (82.9)	
Metformin usage			1.00
Yes	104 (99.0)	104 (99.0)	
No	1 (1.0)	1 (1.0)	
Sulfonylureas usage			0.25
Yes	71 (67.6)	62 (59.0)	
No	34 (32.4)	43 (41.0)	
Insulin usage			0.22
Yes	26 (24.8)	35 (33.3)	
No	79 (75.2)	70 (66.7)	

*Data are presented as mean [standard deviation (SD)] or number (%)*.

*Chi-square test and Fisher's exact test were used for comparison of qualitative variables*.

Table 2 shows the participant's general characteristics and biochemical markers across the tertiles of DDS. ACR (*p* < 0.001), serum albumin (*p* = 0.01), and serum LDL cholesterol (*p* = 0.04) showed a downward trend across the tertiles of DDS.

**Table 2:** General characteristics and biochemical markers of participants across tertiles of dietary diversity score^a^.

**Variable**	**Dietary diversity score tertiles**			
	**Tertile 1**	**Tertile 2**	**Tertile 3**	***P* trend^**b**^**
Age (y)	54.21 ± 6.88	56.52 ± 6.51	55.39 ± 7.67	0.32
Body weight (kg)	72.38 ± 11.26	72.55 ± 12.29	72.55 ± 14.53	0.93
Energy (kcal/day)	1,447.34 ± 240.33	1,471.49 ± 366.00	1,372.45 ± 241.57	0.12
Diabetes duration (y)	7.84 ± 2.10	7.67 ± 2.21	7.23 ± 2.22	0.10
ACR	188.10 ± 136.49	100.17 ± 127.92	88.17 ± 115.55	<0.001
Albumin (mg/dl)	13.59 ± 7.37	11.21 ± 13.24	9.39 ± 8.63	0.01
SBP (mmHg)	125.43 ± 16.94	137.55 ± 121.17	120.70 ± 16.64	0.69
DBP (mmHg)	83.11 ± 13.60	81.54 ± 12.45	79.72 ± 11.24	0.11
FBS (mg/dl)	163.47 ± 48.64	156.80 ± 53.03	161.61 ± 43.09	0.82
HB A1c (%)	8.56 ± 1.27	8.21 ± 1.51	8.26 ± 1.35	0.20
TC (mg/dl)	186.73 ± 33.66	177.36 ± 36.48	176.72 ± 36.31	0.10
TG (mg/dl)	176.43 ± 60.73	150.29 ± 60.46	167.30 ± 62.25	0.38
LDL (mg/dl)	106.43 ± 30.63	99.91 ± 31.22	95.90 ± 31.21	0.04
HDL (mg/dl)	45.04 ± 7.99	46.07 ± 9.26	46.01 ± 10.44	0.53
Creatinine (mg/dl)	0.90 ± 0.15	0.89 ± 0.19	0.90 ± 0.16	0.92
BUN (mg/dl)	15.85 ± 4.95	15.01 ± 3.82	15.58 ± 3.80	0.70
Physical activity^c^ (%)				0.60
Low	24 (34.3)	18 (26.1)	26 (36.6)	
Moderate	25 (35.7)	25 (36.2)	20 (28.2)	
High	21 (30.0)	26 (37.7)	25 (35.2)	
CVD history (%)	14 (20.0)	15 (21.7)	18 (25.4)	0.75
ARB drugs user (%)	35 (50.0)	31 (44.9)	39 (54.9)	0.50
ACEI drugs user (%)	21 (30.0)	22 (31.9)	22 (31.0)	0.98
Beta-blocker drugs user (%)	13 (18.6)	15(21.7)	10 (14.1)	0.50
Metformin user (%)	70 (100.0)	67 (97.1)	71 (100.0)	0.11
Sulfonylurea drugs user (%)	45 (64.3)	43 (62.3)	45 (63.4)	0.98
Insulin user (%)	20 (28.6)	23 (33.3)	18 (25.4)	0.59

*BMI, body mass index; ACR, albumin creatinine ratio; SBP, systolic blood pressure; DBP, diastolic blood pressure; FBS, fasting blood sugar; HB, hemoglobin; TC, total cholesterol; TG, triglyceride; LDL, low-density lipoprotein; HDL, high-density lipoprotein; blood urine nitrogen; CVD, cardiovascular disease; ARB, angiotensin receptor blockers; ACIE, angiotensin-converting enzyme inhibitors*.

a *Data are presented as mean ± SD or number (percent)*.

b *ANOVA test was used*.

c *Chi-square test was used*.

Dietary intakes of participants across tertiles of DDS are presented in Table 3. It was observed that increased DDS was significantly associated with decreased intake of carbohydrate (*p* = 0.04), vitamin E (*p* = 0.02), vitamin C (*p* = 0.01), vitamin B1 (*p* = 0.003), vitamin B2 (*p* ≤ 0.001), vitamin B3 (*p* = 0.001), vitamin B5 (*p* = 0.02), vitamin B9 (*p* ≤ 0.001), sodium (*p* ≤ 0.001), and iron (*p* = 0.009). Moreover, increased DDS was significantly related to increased intake of cholesterol (*p* = 0.001), vitamin B12 (*p* = 0.01), calcium (*p* = 0.004), magnesium (*p* = 0.03), and zinc (*p* = 0.04).

**Table 3:** Dietary intakes of participants across tertiles of dietary diversity score^a^.

**Variables**	**Dietary diversity score tertiles**			
	**Tertile 1** **(*N* = 70)**	**Tertile 2** **(*N* = 69)**	**Tertile 3** **(*N* = 71)**	***P*-value^**b**^**
Protein (gr/day)	47.35 ± 0.56	46.37 ± 0.57	47.23 ± 0.56	0.41
Carbohydrate (gr/day)	250.01 ± 1.78	254.96 ± 1.80	249.01 ± 1.78	0.04
Total fat (gr/day)	33.11 ± 0.68	31.86 ± 0.69	33.91 ± 0.68	0.11
Cholesterol (mg/day)	4.58 ± 0.82	8.97 ± 0.83	6.69 ± 0.82	0.001
Saturated fat (gr/day)	6.16 ± 0.14	6.03 ± 0.14	6.43 ± 0.14	0.11
Vitamin A (RAE/day)	38.58 ± 2.07	38.54 ± 2.09	41.74 ± 2.07	0.46
Vitamin K (μg/day)	13.71 ± 0.52	12.23 ± 0.52	13.30 ± 0.52	0.12
Vitamin E (mg/day)	4.02 ± 0.13	4.35 ± 0.13	3.85 ± 0.13	0.02
Vitamin C (mg/day)	10.69 ± 0.63	11.67 ± 0.64	8.88 ± 0.63	0.01
Vitamin B1 (mg/day)	1.76 ± 0.03	1.64 ± 0.03	1.67 ± 0.03	0.003
Vitamin B2 (mg/day)	1.02 ± 0.01	0.93 ± 0.01	0.96 ± 0.01	<0.001
Vitamin B3 (mg/day)	16.75 ± 0.22	15.69 ± 0.22	15.75 ± 0.22	0.001
Vitamin B5 (mg/day)	2.59 ± 0.07	2.47 ± 0.07	2.32 ± 0.07	0.02
Vitamin B6 (mg/day)	0.77 ± 0.01	0.76 ± 0.01	0.77 ± 0.01	0.85
Vitamin B9 (μg/day)	412.87 ± 8.47	368.23 ± 8.56	370.88 ± 8.47	<0.001
Vitamin B12 (μg/day)	0.11 ± 0.01	0.18 ± 0.01	0.15 ± 0.01	0.01
Sodium (mg/day)	3,811.36 ± 112.47	3,162.67 ± 113.61	3,595.04 ± 112.37	<0.001
Potassium (mg/day)	1,722.02 ± 36.15	1,684.84 ± 36.51	1,716.07 ± 36.12	0.74
Calcium (mg/day)	404.56 ± 5.10	391.06 ± 5.15	415.61 ± 5.10	0.004
Iron (mg/day)	15.20 ± 0.14	14.59 ± 0.14	14.90 ± 0.14	0.009
Phosphorous (mg/day)	930.83 ± 13.60	891.86 ± 13.73	897.76 ± 13.58	0.09
Magnesium (mg/day)	361.52 ± 7.39	339.61 ± 7.46	365.52 ± 7.38	0.03
Zinc (mg/day)	8.08 ± 0.23	7.85 ± 0.23	8.63 ± 0.23	0.04

a *Data are presented as mean ± SE (except for energy intake that presented as mean ± SD)*.

b *ANCOVA test was used*.

Table 4 describes multivariable conditional logistic regression models for the odds of DN according to the total DDS and variety within the food groups. In the crude model, higher adherence (third vs. the first tertile) to the DDS [odds ratio (OR) = 0.24; 95% CI (0.11–0.49)], fruits [OR = 0.13; 95% CI (0.05–0.31)], and vegetables intake [OR = 0.13; 95% CI (0.05–0.37)] was significantly associated with decreased odds of DN. Protein [OR = 0.76; 95% CI (0.38–1.53)], grain [OR = 2.03; 95% CI (0.80–5.13)], and dairy [OR = 1.15; 95% CI (0.57–2.34)] intakes were not associated with odds of DN. After adjusting for potential confounders, an inverse relationship between the odds of DN and total DDS [OR = 0.13; 95% CI (0.05–0.35)], fruits group [OR = 0.05; 95% CI (0.01–0.20)], and vegetables group [OR = 0.09; 95% CI (0.02–0.36)] was observed and the lack of association with protein, grain, and dairy remained.

**Table 4:** Conditional logistic regression models for odds of diabetic nephropathy according to total dietary diversity score and variety within the food groups^a^.

**Dietary diversity score and its components**	**Odds of diabetic nephropathy**			
	**No. cases/controls**	**Crude**	**Model 1**	**Model 2**
**Total dietary diversity score**				
Tertile 1 (<4.42)	53/17	1.00 (Ref)	1.00 (Ref)	1.00 (Ref)
Tertile 2 (≥4.42– <5.51)	26/43	0.19 (0.08–0.43)	0.15 (0.06–0.39)	0.10 (0.03–0.33)
Tertile 3 (≥5.51)	26/45	0.24 (0.11–0.49)	0.21 (0.10–0.46)	0.13 (0.05–0.35)
*P*-trend		<0.001	<0.001	<0.001
**Grains group score category**				
Category 1 (≤0.5)	11/12	1.00 (Ref)	1.00 (Ref)	1.00 (Ref)
Category 2 (>0.5– ≤ 1.0)	25/54	0.41 (0.15–1.12)	0.28 (0.09–0.86)	0.40 (0.11–1.54)
Category 3 (>1.0)	69/39	2.03 (0.80–5.13)	1.52 (0.54–4.29)	3.14 (0.82–12.07)
*P*-trend		0.003	0.008	0.002
**Fruits group score category**				
Category 1 (≤0.5)	45/15	1.00 (Ref)	1.00 (Ref)	1.00 (Ref)
Category 2 (>0.5– ≤ 1.0)	47/45	0.40 (0.19–0.85)	0.34 (0.15–0.79)	0.22 (0.07–0.66)
Category 3 (>1.0)	13/45	0.13 (0.05–0.31)	0.10 (0.04–0.26)	0.05 (0.01–0.20)
*P*-trend		<0.001	<0.001	<0.001
**Dairies group score category**				
Category 1 (≤0.5)	18/24	1.00 (Ref)	1.00 (Ref)	1.00 (Ref)
Category 2 (>0.5– ≤ 1.0)	39/26	1.86 (0.87–3.98)	1.92 (0.86–4.29)	1.86 (0.72–4.80)
Category 3 (>1.0)	48/55	1.15 (0.57–2.34)	1.19 (0.56–2.52)	0.97 (0.41–2.33)
*P*-trend		0.93	0.91	0.47
**Vegetables group score category**				
Category 1 (≤0.5)	26/5	1.00 (Ref)	1.00 (Ref)	1.00 (Ref)
Category 2 (>0.5– ≤ 1.0)	46/26	0.44 (0.15–1.27)	0.36 (0.12–1.10)	0.45 (0.11–1.91)
Category 3 (>1.0)	33/74	0.13 (0.05–0.37)	0.12 (0.04–0.34)	0.09 (0.02–0.36)
*P*-trend		<0.001	<0.001	<0.001
**Proteins group score category**				
Category 1 (≤0.5)	41/29	1.00 (Ref)	1.00 (Ref)	1.00 (Ref)
Category 2 (>0.5– ≤ 1.0)	32/47	0.40 (0.19–0.86)	0.35 (0.15–0.82)	0.26 (0.08–0.78)
Category 3 (>1.0)	32/29	0.76 (0.38–1.53)	0.77 (0.36–1.63)	0.61 (0.24–1.52)
*P*-trend		0.44	0.50	0.26

a *Conditional logistic regression was used*.

*Data are presented as odds ratio [95% confidence interval (CI)]*.

*Crude: Unadjusted model*.

*Model 1: Adjusted for age, body mass index, energy intake, and physical activity*.

*Model 2: Adjusted for confounders in model 1 plus diabetes duration, cardiovascular diseases history, and drug usage (angiotensin receptor blockers; angiotensin-converting enzyme inhibitors, beta-blockers, metformin, sulphonylurea, and insulin)*.

## Discussion

The present case-control study conducted on Iranian women with T2D displayed evidence of a significant inverse association between higher DDS, consumption of fruits and vegetables with the odds of DN. These findings are not unexpected; since a higher fruit and vegetable intake is linked to a more varied diet. Since DDS reflects a comprehensive view of the individual diet, it is a useful indicator for recognizing the association between diet and risk of chronic diseases (26).

Our study illustrated the possible beneficial effects of dietary diversity on renal function and DN. Moreover, this study demonstrated that the consumption of fruits and vegetables may reduce the odds of DN. A cross-sectional study on 110 newly diagnosed T2DM revealed that serum creatinine levels of patients were negatively associated with DDS (16). In a previous population-based cohort study, greater total diet diversity was associated with a 30% lower risk of progression T2D. In addition, in this study, greater diversity in dairy products, fruits, and vegetables was related to the lower incidence of diabetes (13). In another study, Tiew et al. reported that working diabetic patients have a less diversified diet (26). Moreover, Du et al. in a prospective study on Chinese adults suggested that higher fresh fruit consumption was related to the lower risk of diabetes, death, and major vascular complications among diabetic patients (27). Another study proposed that fruits and vegetables appear to be efficient in renal protection in hypertensive and possibly other nephropathies (28). In addition, a meta-analysis of cohort studies concluded that although there was not a significant association between total fruit and vegetable consumption and T2D risk, 2–3 and 2 servings/day of vegetable and fruit, respectively, should be recommended for the prevention of T2D (29).

Despite the recommendation for decreased protein intake in patients with DN, we did not observe a relationship between meat and dairies. In a cross-sectional study on 420 long-standing T2DM, higher intake of vegetable protein was associated with a less prevalence of renal impairments, and theoretical substitution of animal protein with vegetable protein was inversely associated with renal dysfunction among patients with T2DM (30). Moreover, Malhotra et al. reported that diabetic people had higher protein intakes than those without diabetes, and it was associated with an increased risk of ESRD in blacks, not white diabetics. However, whether protein intake raises the risk of ESRD is uncertain (31).

We found a negative association between DDS and serum LDL cholesterol levels but not with other cardiovascular risk factors. A recent study revealed that higher dietary diversity was associated with lower abnormalities of TG and HDL levels (32). Another study by Azadbakht et al. reported that the probability of hypertension, hypercholesterolemia, and high LDL was decreased in the higher quartile of the DDS (33). Furthermore, a cross-sectional study among subjects with metabolic syndrome observed lower serum TG and SBP with greater DDS (15). In contrast, a case-control study in Iran showed a significant positive association between DDS and obesity (34).

Differences in the dietary assessment tools, numbers of food groups, scoring of dietary diversity methods, sample size, and type of study design might cause inconsistent results among some studies. To our knowledge, previous studies were cross-sectional, but this is the first case-control study that investigates the association between DDS and DN.

Several mechanisms might be responsible for the association between DDS and its components with DN odds. The lack of diversity in diet can lead to a loss of variety in intestinal microbiota and lower health outcomes (35). Consumption of diverse foods, especially vegetables and fruits, is optimal for achieving nutrient intakes to promote health (36). Vegetables and fruits are rich sources of vitamin C, carotenoids, potassium, and dietary fiber (37). Their fiber content may exert a favorable effect on renal through bodyweight control, short-chain fatty acid production, reduction of uremic toxins, inflammation, and glycemic control (38). Based on existing evidence, good glycemic control reduces albuminuria and serum creatinine levels (39). Since diabetes has high oxidative potential, the antioxidant content of vegetables and fruits could be useful for reducing vascular complications among these patients (40).

The present study has multiple strengths. First, the cases and controls were selected during the same period from the same place. Second, it was the first study that assessed the association of DDS with DN odds directly. Third, the assessment of dietary intakes was using a validated and reliable FFQ. Nevertheless, we acknowledge some limitations. First, due to the nature of the case-control studies, the recall and selection biases in these studies are not unavoidable. Second, although, we individually matched cases and controls for age and diabetes duration, however, other confounders, such as BMI, were not considered. Third, this study had a relatively low sample size.

## Conclusion

The findings of the present case-control study indicate that higher DDS in individual diets diminished the odds of DN. This association might be due to the two components of DDS, namely, fruits and vegetables. Because of the existing limitations, we suggest further well-controlled prospective studies with larger sample sizes are needed to confirm these findings.

## Data Availability Statement

The raw data supporting the conclusions of this article will be made available by the corresponding author with rational reasons.

## Ethics Statement

The studies involving human participants were reviewed and approved by Ethics Committee of Tehran University of Medical Sciences (Ethic Number: IR.TUMS.REC.1395.2644) and Ethics Committee of Semnan University of Medical Sciences (Ethic Number: IR.SEMUMS. REC.1395.66). The patients/participants provided their written informed consent to participate in this study.

## Author Contributions

MR: manuscript writing. FM, YJ, and MA: study designing and interpretation of analyzed outputs. AJ: data collection. MSY: data analysis. KM: study management and supervision of the final manuscript. KC and LS: critically read and edited the manuscript. All authors contributed to the article and approved the submitted version.

## Funding

This work was supported by the Tehran University of Medical Sciences (Grant Number: 94-04-161-31155).

## Conflict of Interest

The authors declare that the research was conducted in the absence of any commercial or financial relationships that could be construed as a potential conflict of interest. The reviewer PM declared a shared affiliation with two of the authors, MR and FM, to the handling editor at time of review.

## Publisher's Note

All claims expressed in this article are solely those of the authors and do not necessarily represent those of their affiliated organizations, or those of the publisher, the editors and the reviewers. Any product that may be evaluated in this article, or claim that may be made by its manufacturer, is not guaranteed or endorsed by the publisher.

## Abbreviations

ACR: albumin-to-creatinine ratio
BMI: body mass index
BUN: blood urea nitrogen
CKD: chronic kidney disease
Cr: creatinine
DASH: Dietary Approaches to Stop Hypertension
DDS: dietary diversity score
DN: diabetic nephropathy
eGFR: estimated glomerular filtration rate
ESRD: end-stage renal disease
FBG: fasting blood glucose
FFQ: food frequency questionnaire
HbA1c: glycosylated hemoglobin
HDL: high-density lipoprotein
IPAC: physical activity questionnaire
LDL: low-density lipoprotein
TC: total cholesterol
TG: triglycerides
T2D: type 2 diabetes
2hrBG: 2-h post-load blood glucose.
